# Use of point-of-care ultrasound to diagnose spontaneous rupture of fibroid in pregnancy

**DOI:** 10.24908/pocus.v6i1.14757

**Published:** 2021-04-22

**Authors:** Stephen Lammers, Christopher Hong, Jared Tepper, Christy Moore, Cameron Baston, Cara D Dolin

**Affiliations:** 1 Department of Obstetrics & Gynecology, University of Pennsylvania Perelman School of Medicine Philadelphia, PA; 2 Department of Emergency Medicine, University of Pennsylvania Philadelphia, PA; 3 Department of Medicine, University of Pennsylvania Philadelphia, PA

**Keywords:** FAST exam, hemoperitoneum, pregnancy, fibroid, POCUS

## Abstract

**Background:** Complications of fibroids in pregnancy are well known, including postpartum hemorrhage, labor dystocia, and cesarean delivery. Outside of pregnancy and labor, the rare occurrence of spontaneous fibroid rupture has been documented. **Case:** The current case report involves a woman who presented with acute abdominal pain in the third trimester of pregnancy and was found to have spontaneous rupture of a fibroid before the onset of labor. Her initial presentation, diagnosis through use of point-of-care ultrasound, acute surgical management, and postoperative course are described. **Conclusion:** When assessing acute abdominal pain in a pregnant patient, fibroid rupture should be considered despite the absence of prior uterine surgery. Bedside point-of-care ultrasonography is a useful tool for assessment of abdominal pain in the third trimester of pregnancy.

## Introduction

Uterine fibroids are smooth muscle, benign tumors found in 12-25% of reproductive-age women 1. Up to 10% of pregnancies are complicated by fibroids 2.In pregnancy, the presence of fibroids has been associated with an increased risk of adverse pregnancy outcomes including labor dystocia, postpartum hemorrhage, abnormal fetal presentation and cesarean delivery 2, 3, 4.Cumulatively, the presence of fibroids in pregnancy is associated with 10-40% incidence of obstetric complications 5, 6. 

Outside of pregnancy, spontaneous rupture of fibroid and subsequent hypovolemic shock necessitating emergency laparotomy for control of hemorrhage has been reported in young, nulliparous women as well as in post-menopausal women 7, 8, 9, 10. Rare case reports have described spontaneous rupture of fibroids occurring during pregnancy in women without prior myomectomies, notably in the second trimester as well as postpartum 11, 12, 13, 14.

In both pregnant and non-pregnant patients presenting with abdominal pain and concern for active hemorrhage, point-of-care ultrasound (POCUS) has been utilized for triage and management decision making 8, 9, 10, 12.In the present report, we describe the use of POCUS for the evaluation of a patient with a known fibroid and no prior surgical history presenting with acute abdominal pain and concern for active hemorrhage in the third trimester of pregnancy.

## Case presentation

**Figure 1:**
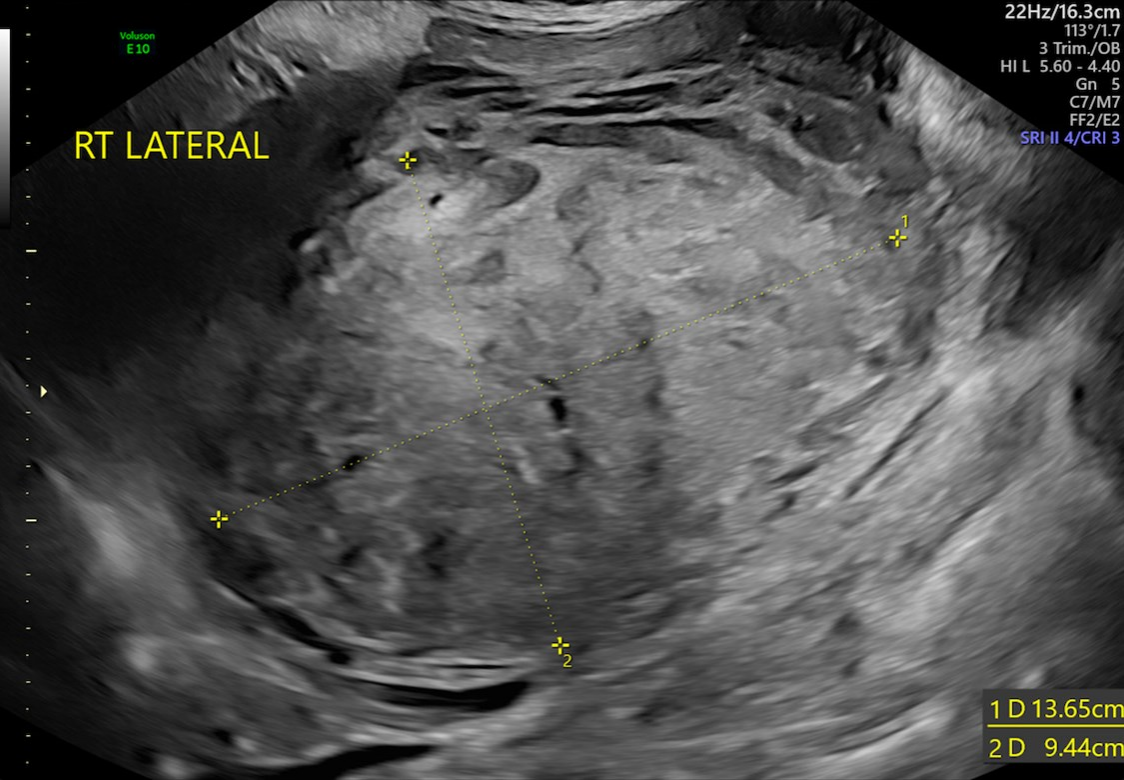
Representative image of a fibroid in the third trimester of pregnancy.

A 28-year-old, gravida 3 para 0, at 35 and 6/7 weeks of gestation presented to a triage unit with worsening abdominal pain. Her subjective history was notable for abdominal cramping the previous evening that progressively worsened through the morning of presentation. She described her pain as constant and sharp with radiation towards her back. She reported regular fetal movement and did not report any leakage of fluid or vaginal bleeding. Her review of symptoms was otherwise negative. Prior to pregnancy, her medical history was notable for a left-sided subserosal fibroid measuring 6.7 x 5.0 x 8.2 cm by transabdominal ultrasound that was identified in a workup for abnormal uterine bleeding. This fibroid then measured 8.4 x 10.2 x 6.1 cm at 20 weeks of gestation and 9.5 x 9.3 x 8.4 cm at 32 weeks of gestation (Figure 1). Her prenatal course was otherwise uncomplicated. Her medical history was unremarkable and prior to pregnancy she took no regular medications. Her surgical history was notable for one prior surgical abortion via dilation and evacuation.

**Figure 2:**
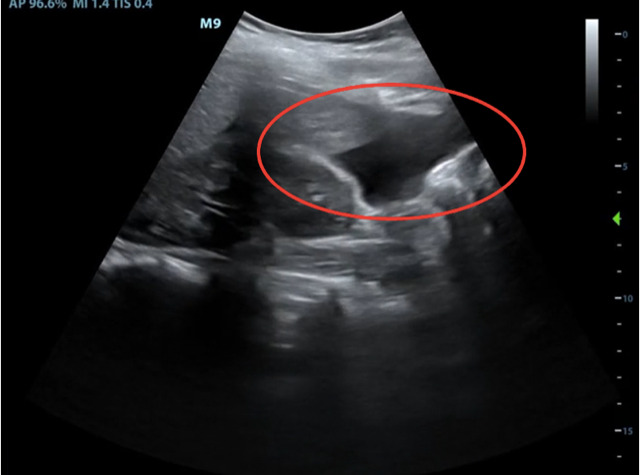
Positive FAST exam of the right upper quadrant. Free fluid is highlighted inferior to the liver with red circle. More anatomy visible in Supplementary Video S1.

**Figure 3:**
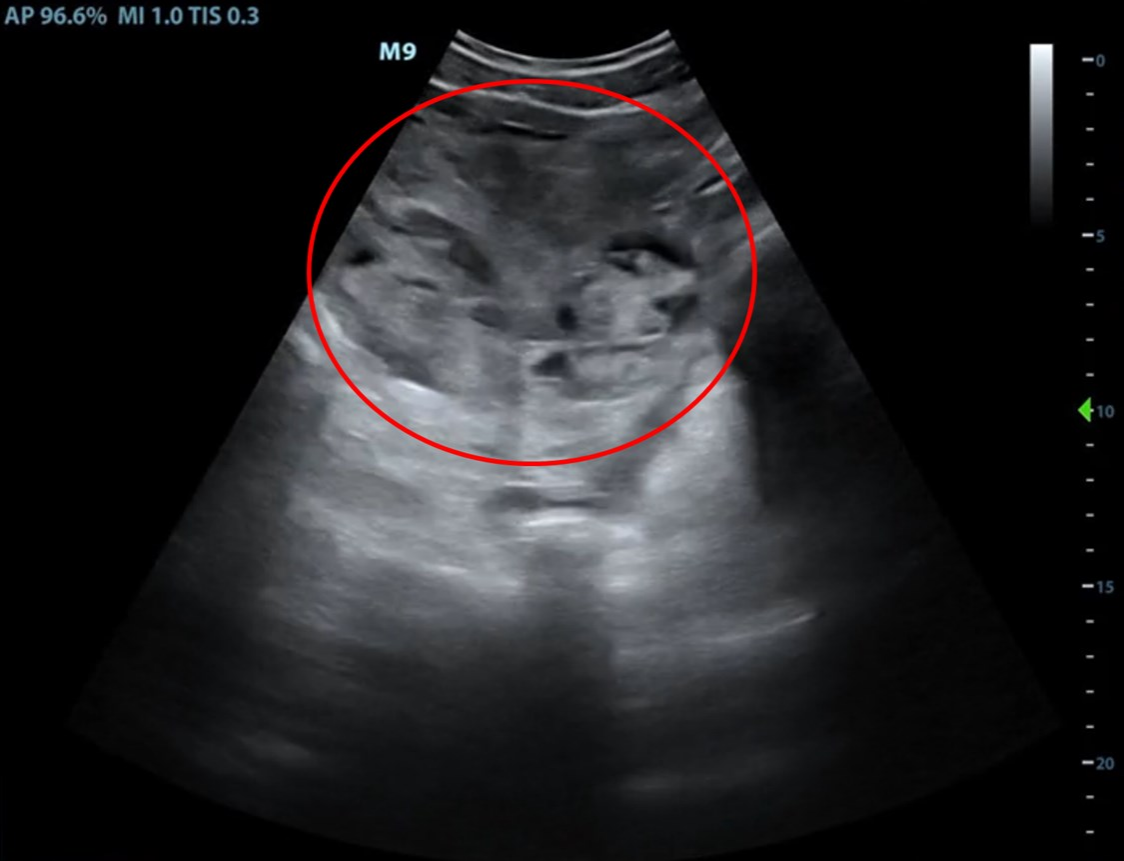
Positive FAST exam of the pelvis. Clotted blood is highlighted in red circle. More anatomy visible in Supplementary Video S2.

 On initial presentation, her vital signs were as follows: pulse of 122 beats per minute, blood pressure of 146/67 mm Hg, respiratory rate of 22/min, and oxygen saturation of 96% on room air. On physical exam, the patient was alert and oriented and in mild distress. Cardiac exam was notable for regular rhythm tachycardia by manual pulse palpation and pulmonary exam was unremarkable. Abdominal exam demonstrated a firm and severely tender abdomen in all four quadrants with rebound tenderness. Cervical exam was 1cm dilation, 50% effacement, and -3 station. On electronic fetal heart monitoring, the fetus had a baseline heart rate of 140 beats per minute, moderate variability, and no accelerations or decelerations over a 20-minute period. On external tocometry, there were regular contractions every 2 minutes. Transabdominal POCUS demonstrated a cephalic fetus and large, bilateral collections of free fluid in the maternal abdomen. Figure 2 (supplemental Video S1) and Figure 3 (supplemental Video S2) show representative findings in the right upper quadrant and pelvis, respectively. Laboratory evaluation showed a hematocrit of 37%, platelets of 415 x10^3^/µL, serum creatinine of 0.7 mg/dL, AST/ALT 22/20 units/L, total bilirubin of 0.5 mg/dL, and LDH 195 U/L. Coagulation studies showed an INR of 0.9, PTT of 26 seconds, and fibrinogen of 700 mg/dL.

**Figure 4:**
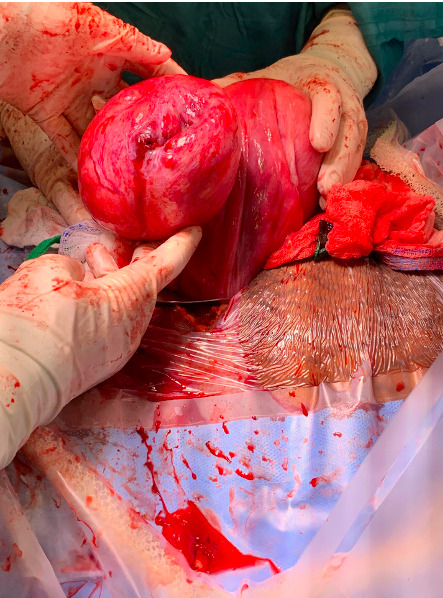
Left sided fibroid with capsular rupture.

In the setting of worsening abdominal pain with positive rebound tenderness and significant free fluid concerning for hemoperitoneum, possible uterine rupture was suspected. The patient underwent an emergency cesarean delivery under general anesthesia. Upon entry into the peritoneum, light brown serosanguinous fluid and dark blood was visualized without evidence of an active hemorrhage. Inspection of the uterus and adnexal structures revealed an 8 to 10 cm uterine fibroid on the left side that distorted the lower uterine segment (Figure 4). No uterine dehiscence or rupture was identified. To avoid a low transverse hysterotomy extending into the fibroid, a midline vertical hysterotomy in a classical fashion was performed. A viable, male infant was then delivered through the hysterotomy. The placenta was removed intact. The uterus was exteriorized and the hysterotomy was then closed in three layers. The fibroid was again inspected and a rupture in the fibroid capsule was appreciated (Figure 4). The serosal capsule was entered and the contents within the fibroid were hemorrhagic, liquefied, and nearly entirely degenerated with minimal fibroid tissue remaining (Supplementary Image 1). Given these findings, the decision was made to proceed with a myomectomy. The fibroid was grasped with a tenaculum and the capsule was dissected from the underlying myometrium using a combination of blunt and electrosurgical dissection to shell out the remaining contents of the fibroid. The defect was then repaired in three layers. The uterus was returned to the abdomen and the remainder of the surgery was completed without complications. The infant had APGAR scores of 3 and 8 at 1 and 5 minutes, respectively. Total estimated blood loss from the procedure was 1800 mL. Intraoperatively, the patient did not require any blood products or vasopressor therapy. Postoperatively, the patient was transferred to the postpartum floor in stable condition. Her postoperative course was notable for acute blood loss anemia with a Hgb nadir of 7.0 g/dL; the patient declined blood transfusion. Otherwise, her postoperative course was uncomplicated and she was discharged home on postoperative day #4 in stable condition. Final pathology resulted as benign leiomyoma.

## Discussion

While it has been established that prior myomectomy is a risk factor for uterine rupture in pregnancy, there have been only a few reports of a ruptured fibroid complicating pregnancy. Spontaneous fibroid rupture postpartum at two days and nine weeks has been reported 13, 14.Similarly, there are also reports of spontaneous rupture of fibroid intrapartumand in the early second trimester at 15 and 20 weeks gestation 11, 12, 15.To date, there is no report of a pregnant woman with known fibroids and no history of myomectomy presenting with spontaneous rupture of fibroid in the third trimester prior to the onset of labor, as we present here. In context of the rarity of this presentation, antepartum knowledge of fibroids is useful for management of abdominal pain in pregnancy.

In women with prior myomectomy and entry into the endometrial cavity, cesarean delivery at 37 weeks of gestation is recommended for the prevention of uterine rupture in labor 16. However, in women without prior myomectomy, there is no consensus on the management of fibroids in pregnancy regarding size, number, and interval growth with the effects on pregnancy and labor outcomes. Concern for fibroids obstructing the birth canal is an indication for cesarean delivery 6.In general, myomectomy in pregnancy is not recommended due to concern for pregnancy loss and hemorrhage 17.Myomectomy in pregnancy has been reported for the treatment of intractable pain that has failed conservative management,torsion,renal compromise secondary to obstruction,and septic necrosis 17, 18, 19, 20, 21.Limited retrospective studies have evaluated pregnancy outcomes in women who undergo first or second trimester myomectomies and while successful pregnancy outcomes have been reported conservative management is recommended and surgery is indicated only on a case-by-case basis 18, 22, 23.

 While the rates of hemorrhage, fetal malpresentation, cesarean delivery, and other adverse obstetrical outcomes associated with fibroids are beginning to be understood, it is not definitively known to what effect myomectomy in pregnancy or planned cesarean delivery decreases the incidence of these outcomes. As this and other case reports suggest, the spontaneous rupture of fibroid in pregnancy is a very rare event. Thus, in a pregnant woman with fibroids, the risks associated with myomectomy in pregnancy or planned cesarean delivery likely outweigh the benefits of preventing spontaneous rupture of fibroid and other adverse obstetric outcomes associated with fibroids in labor.

Importantly, this case demonstrates the usefulness of point-of-care transabdominal ultrasonography (POCUS) in the evaluation and management of acute abdominal pain in pregnancy. Ultrasonography is a useful diagnostic tool for the detection of hemoperitoneum, visualized as free fluid in the abdomen or pelvis, especially in the setting of blunt and penetrating abdominal trauma 24, 25.In a patient with acute blunt or penetrating abdominal trauma, Focused Assessment with Sonography in Trauma (FAST) exam has a reported sensitivity of 69-98% for the detection of hemoperitoneum, with the higher ranges reported for patients with hypotension following trauma 26, 27.The specificity of FAST exam for free fluid in this patient population is reportedly 94-100% 26.Follow up reviews have found that in hemodynamically stable patients the sensitivity may be as low as 22-28%, therefore serial exams and/or follow up imaging are recommended 28.

Among pregnant patients presenting with acute blunt or penetrating abdominal trauma, FAST exams have a sensitivity of 61-85% and specificity of up to 99% for the detection of intraperitoneal free fluid; this is similar to the non-pregnant population 26, 27, 29, 30, 31. For image acquisition, obtaining the pelvic views for evaluating free fluid in the pouch of Douglas may be more difficult secondary to the gravid uterus 26. Nevertheless, there are no recommendations that the FAST exam be modified regarding how to obtain the standard FAST exam images when evaluating a pregnant patient with abdominal trauma. Compared to non-pregnant patients, ultrasound for the identification of hemoperitoneum may be associated with higher false positives secondary to increased physiologic free fluid 29. However, specificity and negative predictive value for ruling out free fluid appear to be similar between pregnant and non-pregnant patients. Thus, while pregnant patients may have increased physiologic free fluid at baseline, when presenting with abdominal trauma and FAST scan is negative, this is reassuring for ruling out active bleeding and further diagnostic workup may be warranted pending overall maternal and fetal status. 

In the present case, POCUS showed significant free fluid in the pelvis in the bilateral lower quadrants concerning for hemoperitoneum. The differential diagnosis included uterine rupture, placental abruption, fibroid rupture, fibroid necrosis, rupture of adnexal mass, as well as vascular and gastrointestinal etiologies. Although uterine rupture and placental abruption are not usually diagnosed with ultrasound, POCUS in the present case allowed for the rapid identification of intra-abdominal hemorrhage with reasonable certainty. Whereas POCUS did not rule in or out specific etiologies in this case, it appropriately identified a concerning intra-abdominal process necessitating urgent intervention. While the patient did not have a history of blunt or penetrating abdominal trauma, her overall clinical presentation was concerning for acute hemorrhage. In the context of tachycardia, rebound tenderness, and ultrasonographic signs of intra-abdominal bleeding, the decision was made to proceed with emergency exploratory laparotomy and delivery. With the use of FAST exam, intraabdominal bleeding was identified without requiring transport to CT scanning or serial lab monitoring, and she was transported to the operating room for stabilization, likely preventing further clinical deterioration. Ultrasound was therefore a useful tool in the present case for the urgent triage of a pregnant patient presenting with signs and symptoms of an acute abdomen potentially requiring surgical intervention.

Outside of obstetrics, the use of POCUS for the evaluation of unstable patients has been utilized in the fields of emergency medicine, anesthesia, and critical care. For example, the use and interpretation of bedside ultrasound are training requirements in these fields 32, 33, 34. Additionally, the FAST exam skillset is readily taught in general surgery residency in order to appropriately evaluate patients with trauma/critical illness and formal training for POCUS has been developed by the American College of Surgery 35, 36. Furthermore, POCUS skillsets are becoming widely adopted throughout medicine as the American Association of Family Physicians, the American Board of Anesthesiology, the Society of Critical Care Medicine, the American College of Emergency Physicians, as well as the Society for Hospital Medicine have all developed formal training curricula for POCUS 37, 38, 39, 40, 41. The American College of Physicians has also committed to developing curricula, guidelines, and training for POCUS, further demonstrating the rapid uptake of these skillsets throughout clinical medicine 42. However to date, there are no formal training requirements for emergency POCUS skills for obstetrics and gynecology residents.

As the present case highlights, POCUS was vitally important for the evaluation and management of an obstetrical patient with an urgent clinical condition. Given that POCUS is a readily accessible and inexpensive imaging modality, formal training in these techniques like FAST exams should be included in obstetrics and gynecology residencies. Evaluation of obstetrical parameters like fetal growth and cervical length as well as gynecological assessment of the adnexa and endometrium are ultrasonographic skillsets taught during training. Combining basic familiarity in gynecological evaluation of fibroids, for example, along with emergency POCUS skills would allow for further ability to develop a differential and management plan for patients presenting with acute abdominal pain. With this basic familiarity in use of ultrasound, training in POCUS would not require significant burden regarding use of equipment or availability of machines. Obstetrics and gynecology residents will encounter patients with urgent clinical conditions in which POCUS may be helpful, such as in patients with ruptured ectopic pregnancy, postpartum hemorrhage, and abdominal trauma in pregnancy. Beyond the abdominal cavity, POCUS may be useful for the cardiothoracic systems as well for the evaluation of peripartum shortness of breath, post-operative hypoxia/fever, and other clinical presentations in which evaluation of the lung, pleura, and pericardial spaces may be beneficial for the triage of critically ill patients 43, 44. Given that obstetrics and gynecology residents already receive training in basic obstetrical ultrasound technique, the addition of formal training in selected emergency POCUS techniques will further serve their ability to appropriately triage patients with critical illness that they may encounter. 

In review, this case highlights the importance of considering spontaneously ruptured fibroid when triaging a pregnant woman with acute onset of severe abdominal pain, even without a history of prior myomectomy. The present case also highlights the usefulness of POCUS to aid in the medical decision making when evaluating a pregnant patient with severe abdominal pain. When an acute surgical abdomen is suspected in pregnancy the FAST exam is a quick and useful tool during triage. Obstetrics and gynecology residency programs should consider implementation of formal training in POCUS.

The patient consented to the use of de-identified information as well as the operative images. She provided full verbal consent for the publication with the understanding that while all information will be de-identified, absolute anonymity cannot be guaranteed.

## Disclosures

The authors have no conflicts of interest to declare. 

## Supplementary Material

Video S1 Positive FAST exam of the right upper quadrant.

Video S2 Positive FAST exam of the pelvis.
